# Listeria-based immunotherapy directed against CD105 exerts anti-angiogenic and anti-tumor efficacy in renal cell carcinoma

**DOI:** 10.3389/fimmu.2022.1038807

**Published:** 2022-11-11

**Authors:** Mariam Oladejo, Hong-My Nguyen, Ashok Silwal, Britney Reese, Wyatt Paulishak, Maciej M. Markiewski, Laurence M. Wood

**Affiliations:** Department of Immunotherapeutics and Biotechnology, Texas Tech University Health Sciences Center, Abilene, TX, United States

**Keywords:** listeria-based vaccine, immunotherapy, CD105, renal cell carcinoma, vaccines, *Listeria monocytogenes*, tumor-associated vasculature

## Abstract

Targeting tumor-associated angiogenesis is currently at the forefront of renal cell carcinoma (RCC) therapy, with sunitinib and bevacizumab leading to increased survival in patients with metastatic RCC (mRCC). However, resistance often occurs shortly after initiation of therapy, suggesting that targeting the tumor-associated vascular endothelium may not be sufficient to eradicate RCC. This study reports the therapeutic efficacy of a Listeria (Lm)-based vaccine encoding an antigenic fragment of CD105 (Lm-LLO-CD105A) that targets both RCC tumor cells and the tumor-associated vasculature. Lm-LLO-CD105A treatment reduced primary tumor growth in both subcutaneous and orthotopic models of murine RCC. The vaccine conferred anti-tumor immunity and remodeled the tumor microenvironment (TME), resulting in increased infiltration of polyfunctional CD8^+^ and CD4^+^ T cells and reduced infiltration of immunosuppressive cell types within the TME. We further provide evidence that the therapeutic efficacy of Lm-LLO-CD105A is mediated by CD8^+^ T cells and is dependent on the robust antigenic expression of CD105 by RCC tumor cells. The result from this study demonstrates the safety and promising therapeutic efficacy of targeting RCC-associated CD105 expression with Lm-based immunotherapy.

## Introduction

Kidney cancer is one of the top 10 most common cancers in the United States, with renal cell carcinoma (RCC) accounting for over 90% of all kidney cancer cases (1). In advanced stages, response to chemotherapy and radiotherapy is limited, leading to increased mortality in patients with metastatic disease (1–3). Anti-angiogenic tyrosine kinase inhibitors (TKIs) have been approved as first-line treatments in RCC, with their proposed mechanism of action to be disruption of signaling in the VEGF/VEGFR2 pathway (4–6). Unfortunately, therapeutic resistance often occurs shortly after initiation of therapy due to aberrant activation of several pathways, including the TGF-β/CD105 signaling axis (7–13).

CD105 (Endoglin) is a transmembrane glycoprotein that serves as a co-receptor for the TGF-β signaling cascade (14). CD105 is enriched on vascular endothelial cells and is commonly expressed on rapidly proliferating endothelial cells, making it an attractive target for anti-angiogenic therapy (13). However, in RCC, CD105 is expressed on both the tumor-associated vasculature and the tumor cells (15, 16). In fact, tumoral CD105 is characterized as a cancer stem cell marker and serves as an independent prognostic factor (15, 17).

Due to the elevated expression of CD105 by tumor-associated vasculature, several preclinical studies have targeted CD105 with both active and passive immunotherapy. Each of these methods demonstrated promising anti-angiogenic and anti-tumor efficacy in murine breast cancer models (18–20). However, a humanized monoclonal antibody against CD105, TRC105, showed only limited improvement in survival in patients with mRCC (21, 22). Due to the limited efficacy of passive immunotherapeutic targeting of CD105, an active immunotherapeutic approach, as demonstrated in preclinical models of breast cancer with Listeria-based vaccines, may provide a more promising therapeutic avenue to target CD105 in mRCC (19).

Lm-based vaccines are active immunotherapies that exploit the ability of the gram-positive intracellular bacterium, *Listeria monocytogenes* (Lm), to infect antigen-presenting cells, escape phagolysosomal destruction using hemolytic Listeriolysin O (LLO), and replicate in the cytosol where it delivers antigenic cargo to elicit tumor-specific CTL-mediated immune responses (23, 24). Further, Lm-based vaccines can modulate the TME to promote the recruitment of anti-tumor T cells, reduce infiltration of regulatory T cells and mitigate the immunosuppressive functions of myeloid-derived suppressor cells (MDSCs) (25–28). Therefore, Lm-based vaccines provide an efficacious platform that reduces immunosuppression within the TME and breaks tolerance to tumor-associated antigens (TAAs).

In this current study, we describe the successful targeting of tumoral and endothelial CD105 in a murine model of RCC. Lm-LLO-CD105A treatment reduced tumor growth in both subcutaneous and orthotopic models in a CD8^+^ T cell-dependent manner. The efficacy of Lm-LLO-CD105A was associated with increased infiltration of polyfunctional (i.e, IFN-γ, TNF-α, and IL-2 producing) CD8^+^ and CD4^+^ T cells and reduction in immunosuppression within the TME, and reduced tumor vascularization.

## Materials and methods

### TCGA data analysis

The TCGA kidney cancer data was assessed and modified from the Human Protein Atlas (https://www.proteinatlas.org/)

### Mice

Male Balb/c (6-8weeks old) mice were obtained from Jackson Laboratories or Envigo and housed at the Laboratory Animal Resource Center (LARC) of Texas Tech University Health Sciences Center (TTUHSC). All mouse experiments were performed in accordance with the regulations provided by the Institutional Animal Care and Use Committee of TTUHSC.

### Cell lines and culture

Murine Renca cell line (CRL-2947) was purchased from American Type Culture Collection (ATCC, Manassas,VA). Renca cells expressing luciferase (Renca-Luc) were generated by transduction of a clonal population of Renca with lentiviral particles co-expressing luciferase and GFP (Gentarget, San Diego, CA). Stable luciferase-expressing cells were subsequently selected by puromycin selection followed by Fluorescence Activated Cell Sorting (FACS).

CD105 deficient Renca cells (Renca-CD105KD) were generated using a CRISPR-Cas9 vector (Origene, Rockville, MD) with a guide RNA directed against the first exon of the *Cd105* gene. Successfully edited cells were clonally selected following FACS sorting for GFP-positive cells. All cells were cultured in RPMI 1640 media supplemented with 10% Fetal Bovine Serum and 1% Penicillin-Streptomycin and kept at 37°C and 5% CO_2._

### *In vitro* infectivity and cytotoxicity assays

For infectivity assay, 1x10^4^ Renca cells were seeded into each well of 96 well plates 24 hours before infection. Subsequently, cells were infected in triplicates with varying MOIs of Lm-LLO-CD105A or Control Lm for 3 hours. Cells were washed with sterile 1XPBS, and gentamicin treatment (50ug/ml-to kill extracellular *Listeria*) was performed for 1 hour. Cells were then washed and lysed in sterile water and plated on Bacto heart infusion (BHI) plates containing streptomycin (34ug/ml) and chloramphenicol (34ug/ml) to determine the colony-forming units of invaded cells.

For the cytotoxicity assay, 5x10^3^ Renca cells were infected with Lm-LLO-CD105A, or Control Lm as described above in the infectivity experiment. 3 hours post-infection, cells were washed and cultured in media containing 5ug/ml of gentamicin (to remove the extracellular *Listeria*) for 48 hours. After 48 hours, cell death was analyzed by Sulforhodamine B viability assay, following the manufacturer’s protocol.

### Western Blotting

To prepare protein lysates, cells and tissue suspensions were lysed with RIPA buffer supplemented with protease inhibitors. Lysates were then mixed with LDS sample loading buffer and reducing agent and subjected to gel electrophoresis with a 4-12% Bis-Tris polyacrylamide gel. After separation, proteins were transferred to a PVDF membrane followed by overnight incubation at 4^0^C with the following primary antibodies; Biotin anti-mouse CD105(Biolegend, clone MJ7/18), Biotin rat anti-mouse CD31(BD, clone 390), B-actin rabbit mAb (cell signaling, #4970), or GAPDH rabbit mAb (cell signaling, #5174). The next day, blots were incubated with Streptavidin-HRP as secondary antibody for CD105 and CD31 or peroxidase-conjugated goat anti-rabbit antibody (Invitrogen, #32460) as secondary antibody for B-actin and GAPDH. Signal was developed with enhanced chemiluminescence (Thermo Scientific, Waltham, MA, USA) and visualized using a UVP imager.

### Real-time quantitative polymerase chain reaction

For *Cd105* qPCR, RNA was extracted from the Renca cell line, normal kidney tissues, and subcutaneous and orthotopically generated tumors with an RNeasy Mini kit (Qiagen, Germany). For *Cd31* qPCR, RNA was extracted from tumors at the end of the longitudinal tumor load experiment. RNA was converted to cDNA with a High-capacity cDNA Reverse transcription kit (Applied Bioscience, USA). cDNA was then subjected to qPCR analysis with the following primers.*Cd105FOR:5’CAGCCAAAGTCTGCAATCAGG, Cd105REV:5’GCTACTCAGGACAAGATGGTCG.*

*Cd31FOR:5’CCAAAGCCAGTAGCATCATGGTC* and

*Cd31REV:5’ GGATGGTGAAGTTGGCTACA*. Gene expression was normalized to *18s* rRNA.

### Tumor load study

For subcutaneous tumor studies, male Balb/c mice (n=5-16/group) were injected subcutaneously (s.c) with 1x10^6^ Renca cells in the hind flank. After tumors became palpable, mice were immunized intraperitoneally with PBS or 2x10^8^ CFU of either Lm-LLO-CD105A or Lm-LLO-OVA (Control Lm) once weekly for three weeks. Tumor size was measured by calipers every other day, and the tumor volume was calculated as (length x width^2^)÷2 (29). At the end of the experiment, tumors were excised, weighed, and processed for downstream analysis.

For orthotopic tumor studies, male Balb/c mice (n=10/group) were injected with 5x10^4^ of Renca-Luc in the left kidney as previously described (30). Three days post tumor implantation, mice were immunized as described in the subcutaneous tumor study. Tumor progression was monitored by bioluminescence detection with D-luciferin (150mg/kg, PerkinElmer, Waltham, MA.) and acquired with an *in vivo* imaging system (IVIS, PerkinElmer). 21 days after the first vaccination, mice were euthanized, and tumors and lung metastases were collected for further analysis.

### Hemoglobin assay

Male Balb/c mice (n=5/group) were implanted with 1x10^6^ Renca cells mixed with Matrigel (Corning, NY) (100ul of PBS containing 1x10^6^ Renca + 200ul of Matrigel) subcutaneously in the hind flank. Mice were vaccinated with either 2x10^8^ CFU/mouse of either Lm-LLO-CD105A or Control Lm on days 5,12 and 19. Matrigel plugs were removed on day 21 and added to 10 ml of RPMI media supplemented with 10%FBS and 1% Penicillin/Streptomycin and homogenized to obtain a cell suspension. The resulting suspension was centrifuged, and the pellet was lysed with 1ml of ACK lysis buffer for 5 minutes at room temperature. The resultant RBC lysates were subjected to hemoglobin analysis with ThermoScientific Colorimetric Hemoglobin Detection Kit.

### Lymphocyte depletion experiments

CD8^+^ T cells were depleted in subcutaneous tumor-bearing mice by injecting mice with 0.2mg of anti-CD8a (clone 2.43, BioXcell, CT) on days 2,4,7,10,13,16 and 19 post-tumor implantation. For this experiment, four groups were included: group 1 received Phosphate Buffered Saline (PBS) as the control, group 2 received Lm-LLO-CD105A+ PBS, group 3 received Control Lm +PBS and group 4 received Lm-LLO-CD105A+ anti-CD8. In the orthotopic model, CD8^+^T cells were similarly depleted on days 1,3,6,9,13, and 17 post-tumor implantations. In this model, we maintained three experimental groups: group 1 received Control Lm+PBS, group 2 received Lm-LLO-CD105A+PBS, and group 3 received Lm-LLO-CD105A+ anti-CD8a. Tumor load studies were then carried out as previously described.

### Tumoral CD105 dependence study

A CD105 deficient Renca cell line (Renca-CD105KD) was generated using CRISPR-Cas9 to target exon 1 of murine *Cd105.* After confirmation of knockdown, the cell number was titrated by *in vivo* growth assay to determine the number of Renca-CD105KD cells that will generate an equal-sized tumor as the Renca-Control. We determined that 1.2x10^6^ cells of Renca-CD105KD and 1x10^6^ of Renca-Control were sufficient for therapeutic studies. Subsequently, respective cell lines were injected into male Balb/c mice (n=5/group). After tumors became palpable, mice were immunized once weekly for three weeks with either Lm-LLO-CD105A or Control Lm (i.p). Tumor volume was measured every other day. At the endpoint, tumors were excised, weighed, and processed into single-cell suspensions for further analysis.

### Intracellular cytokine staining and flow cytometry

Tumors and spleens from treatment and control groups were processed into single-cell suspensions. 2x10^6^ of each tumor suspension was seeded into 96 well plates and stimulated with a T-cell stimulation cocktail with brefeldin A (Biolegend, San Diego, CA, USA) according to the manufacturer’s protocol. Stimulated cells were surface-stained with PerCP-Cy5.5-CD4 (clone GK 1.5), Pe-Cy7-CD8-(clone 53-6.7), AF700-CD62L(clone MEL-14), APC-Cy7-TCR-(clone H57-597), and intracellularly stained with PE-IFN-γ (clone XMG1.2), FITC-TNF-*α* (clone MP6-XT22), and APC-IL-2 (clone JES6-5H4). To determine the myeloid and Treg populations, non-stimulated cells were surface stained with PercP-Cy5.5-Gr-1(clone RB6-8C5), APC-CD11b (clone M1/70), BV605-CD4 (clone GK1.5), APC-Cy7-TCRβ (clone H57-597), and intracellularly stained with PE-Foxp3 (clone 150D). Zombie Aqua dye was used to discriminate live cells from dead cells. Data was collected with BD LSR Fortessa (BD biosciences, MD, USA), and results were analyzed using Flowjo software (Tree star, Ashland, OR, USA). Antibodies were purchased from Biolegend and used at a dilution factor of 1:100 for intracellular stains and 1:200 for surface stains.

### IFN-γ Elispot

The 96 well plate filtration plates (Millipore, Bedford, MA) were coated with 15ug/ml of rat anti-mouse IFN-γ antibody (clone AN18, MABTECH, Mariemont, OH) in 100ul of PBS and incubated overnight at 4°C. The next day, the wells were washed and blocked with RPMI media supplemented with 10%FBS and 1% Penicillin/Streptomycin. Pooled splenocytes from either Control Lm treatment group or the Lm-LLO-CD105A treatment group were stimulated with 10ug/ml of CD105A peptide EGVSGHKEAYILRILPGSEA. After overnight incubation in pre-coated plates, the plates were washed, followed by incubation with 2ug/ml of IFN-γ antibody (clone R4-6A2, MABTECH) in 200ul for 2hrs. After washing, 100ul of a 1:500 streptavidin-HRP was added and incubated for 1hr at room temperature. Spots were developed by adding 200ul of filtered substrate after washing and incubating at room temperature for 20 minutes. Color development was stopped by washing extensively in tap water and spot-forming cells (SFC) were counted with a dissecting microscope.

### Safety studies

For wound healing studies, Balb/c mice (n=10/group) were immunized once weekly for three weeks with 2x10^8^CFU of Lm-LLO-CD105A or Control Lm (i.p). In the fifth week, wound healing assay was conducted, as previously discussed (31). Briefly, mice were anesthetized, hair was removed by shaving, and skin was sterilized with povidone-iodine. Two circular 5mm wounds were punched on either side of the back using a sterile biopsy punch tool (Integra Miltex, Princeton, NJ, USA). Gap closure rate and average time to closure were monitored. The gap was deemed completely closed when no visible scab was detected. At the experimental endpoint, blood was collected from mice, incubated at 4°C, and centrifuged. The resultant serum was subjected to ELISA for IL-2, IFN-γ, and IL-6 by using BioLegend’s ELISA standard sets, according to manufacturers’ protocol.

### Immunofluorescence and H&E staining

For immunofluorescence, frozen tissue sections 5-um thick were stained with anti-mouse CD31 (Clone 390; BD Pharmingen, Franklin lakes, NJ, USA), CD105 (MJ7/18; Biolegend), VEGFR2 (D5B1; Cell Signaling Technology, Danvers, MA, USA). Texas Red and AF488 conjugated antibodies were used as secondary reagents. Staining in at least 10 fields per section was quantified with Nikon Elements Advanced Research Image Analysis software. Data are expressed as the area occupied by CD31 or VEGFR2 positive cells. For safety studies, 5um thick paraffin-embedded kidney and lung sections were analyzed for tumor-infiltrating lymphocytes by H&E staining as previously described (32).

### Statistical analysis

All results are presented as student t-tests except for lymphocyte depletion studies in the orthotopic model, which is shown as Mann-Whitney U test. Outlier analysis was carried out with the rout method and significant outliers removed. All data were analyzed using GraphPad prism software, and significant p values were depicted in the figures as follows, *P<0.05, **P<0.01, ***P< 0.001, ns >0.05. Error bars are shown as SEM.

## Results

### CD105 is expressed on tumor cells and vasculature in RCC

**Figure 1:**
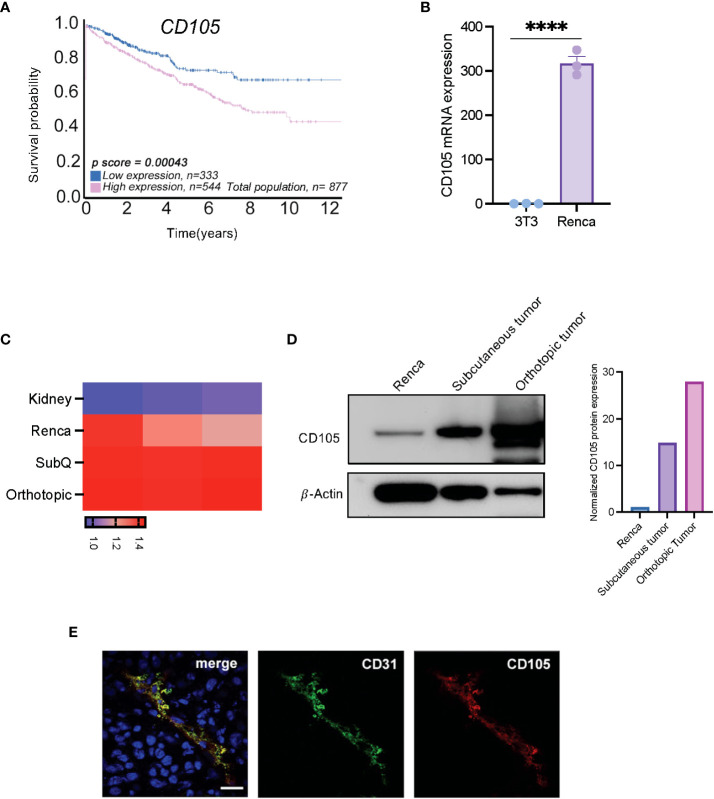
CD105 is highly expressed in RCC, and it correlates with a poor prognosis. **(A)** Overall survival probability of patients expressing a high level of CD105 as compared with those with lower CD105 expression, cutoff = 35.74 fragments per kilobase of transcript per million reads (FPKM). **(B)** mRNA expression of CD105 in NIH-3T3, and **(C)** Renca cell line, normal kidney, and in vivo generated tumors. **(D)** Protein expression of CD105 on Renca cells and tumors derived after subcutaneous and orthotopic implantation of Renca cells in male mice. **(E)** Immunofluorescence visualization of the expression of CD105 and CD31 by Renca tumor tissues **** P < 0.0001. error bar is shown as mean ± SEM.

Since the implication of tumor cell-expressed CD105 has been described, numerous studies have demonstrated its expression on selected cancer cell lines, including human RCC cell lines (17, 33–36). Using data from The Cancer Genome Atlas (TCGA) made available through the Human Protein Atlas (https://www.proteinatlas.org/), we initially verified the prognostic value of CD105 in human RCC; this data showed that patients that have high expression of *CD105* demonstrated an overall lower survival probability (**Figure 1A**).

To confirm that the expression of CD105 is conserved in the murine RCC cell line (Renca), we assayed the expression of CD105 in Renca cells, the non-transformed murine fibroblast cell line, NIH-3T3, and *in vivo* generated Renca tumors. qPCR analysis showed that *Cd105* is expressed to a higher level in the Renca cell line as compared to NIH-3T3 (**Figure 1B**). Further, Renca tumor cells have significantly higher expression of *Cd105* in comparison with the healthy kidney and the expression of this antigen was further increased upon subcutaneous and orthotopic implantation, likely due to vascularization of the tumors (**Figure 1C**). Western blot analysis confirmed this increasing trend in CD105 protein expression after *in-vivo* implantation (**Figure 1D**). To segregate the tumor expression of CD105 from the tumor vasculature-associated expression, we carried out immunofluorescence analysis on the frozen tissue section obtained from subcutaneously implanted tumors. The tumor vasculature-associated expression of CD105 corresponded with the expression of CD31, a pan endothelial cell marker (**Figure 1E**). Altogether, these finding shows the expression of CD105 by both the tumor cells and the vasculature in RCC.

### Lm-LLO-CD105A controls tumor growth in syngeneic models of RCC

**Figure 2:**
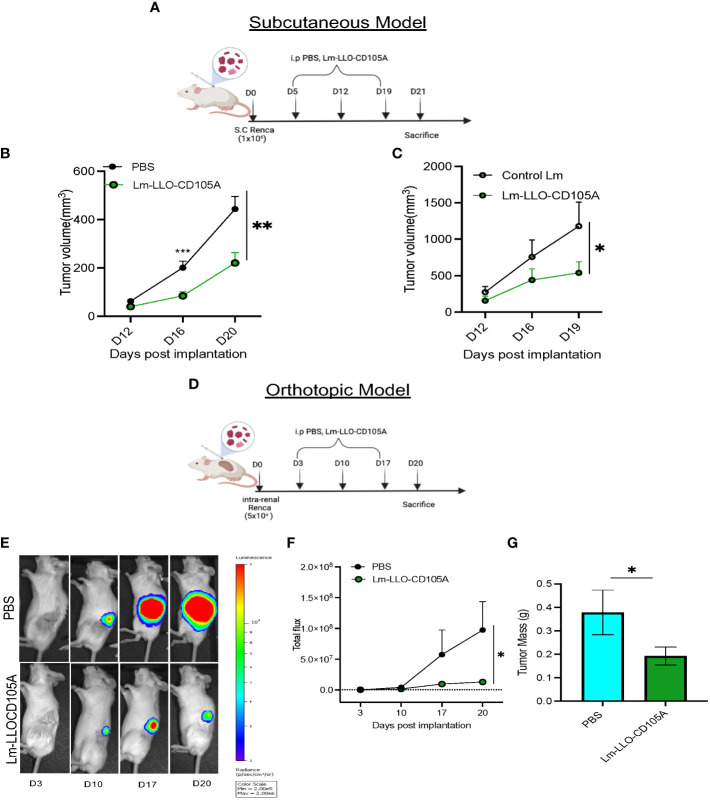
Lm-LLO-CD105 controls RCC tumor growth. **(A)** Vaccination schedule for subcutaneous tumor challenge. **(B, C)** Longitudinal tumor growth curve of subcutaneous tumor challenge (PBS n=16, Control Lm n=5, Lm-LLO-CD105A n=5-10). **(D)** Vaccination schedule for orthotopic tumor challenge. **(E)** Representative bioluminescence imaging of kidney tumor burden. **(F)** Graphical representation of longitudinal luciferase flux. **(G)** Final tumor mass (PBS n=10, Lm-LLO-CD105A n=10). Data were analyzed using unpaired t-test. *P < 0.05, **P < 0.01, ***P< 0.001. All error bars are shown as mean ± SEM. Vaccination schedule depiction was made in BioRender.

To assess the therapeutic potential of a Listeria-based vaccine targeting CD105, Lm-LLO-CD105A, Renca cells were implanted s.c into Balb/c mice, followed by therapeutic vaccinations as depicted (**Figure 2A**). Tumor growth and progression were significantly reduced in the group that received the Lm-LLO-CD105A vaccine as compared to the PBS group (**Figure 2B**). In a separate experiment, Lm-LLO-CD015A also demonstrated significant tumor control in comparison with a Control Lm (**Figure 2C**).

Since the s.c model may not sufficiently recapitulate the TME of RCC (37), we examined the efficacy of Lm-LLO-CD105A against orthotopically-implanted tumors. Three days post-implantation, vaccination was commenced as depicted (**Figure 2D**). Significantly reduced bioluminescence flux (**Figures 2E, F**) and final kidney weight in the Lm-LLO-CD105A vaccine group was observed in comparison to the vehicle (**Figure 2G**). In summary, Lm-LLO-CD105A significantly controls tumor growth in both subcutaneous and orthotopic models of RCC.

### Lm-LLO-CD105A controls tumor angiogenesis

**Figure 3:**
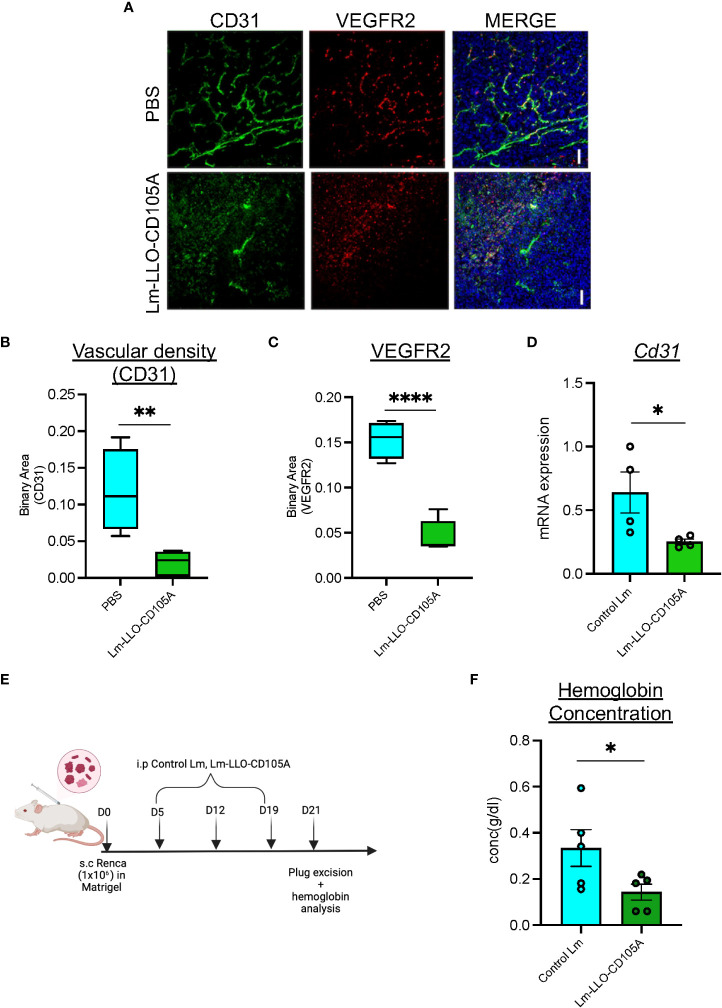
Lm-LLO-CD105 controls tumor angiogenesis. **(A)** Representative images of immunofluorescence-stained sections of PBS control tumors and Lm-LLO-CD105A vaccinated tumor. Slides were stained with antibodies against VEGFR2 and CD31. Scale bar 50um. **(B)** Quantitative vascular density (CD31) of immunofluorescence-stained sections. **(C)** Quantification of VEGFR2 stained immunofluorescence sections **(D)** Relative mRNA expression of CD31 between Control Lm and Lm-LLO-CD105 treated tumors. **(E)** Experimental schema for hemoglobin assay **(F)** Hemoglobin quantification in tumor tissues of Control Lm and Lm-LLO-CD105 vaccine treated tumors. (n=4-5/group) Data were analyzed using unpaired t-test. *P < 0.05, **P < 0.01, **** P<0.0001 . All error bars are shown as mean ± SEM. Experimental schema was made in BioRender.

Studies demonstrating the clinical and preclinical efficacy of CD105-targeting therapies have reported its anti-angiogenic benefits (18, 38). To determine whether Lm-LLO-CD105A can disrupt RCC-associated tumor vasculature, the expression of vascular endothelium-specific proteins CD31 and VEGFR2 was analyzed in tumors from Lm-LLO-CD105A-treated mice (39). CD31 and VEGFR2 positive vessels were significantly reduced in the Lm-LLO-CD105A vaccine-treated group compared to the vehicle group (**Figure 3A**). This correlated to a reduction in vascular density represented by reduced CD31 expression (**Figure 3B**). Similarly, we found a significant reduction in the expression of VEGFR2 (**Figure 3C**). Further, both western blot and qPCR analysis showed a reduction in CD31 protein and *Cd31* mRNA expression in the Lm-LLO-CD105A treatment group compared to the control groups (**Figure 3D** and **Supplementary Figure S2**). As a confirmatory measure of tumor vascularization, tumor-specific hemoglobin concentration was measured (**Figure 3E**). In comparison with the Control Lm-treated group, tumors from Lm-LLO-CD105A treated mice demonstrated a significantly reduced hemoglobin concentration (**Figure 3F**). The significantly reduced CD31 positive vessels, CD31 protein and mRNA expression, and hemoglobin concentration suggest that Lm-LLO-CD105A effectively targets RCC-associated vasculature, contributing to its therapeutic efficacy.

### Lm-LLO-CD105A modulates TME by increasing T cell polyfunctionality and reducing immunosuppression

**Figure 4:**
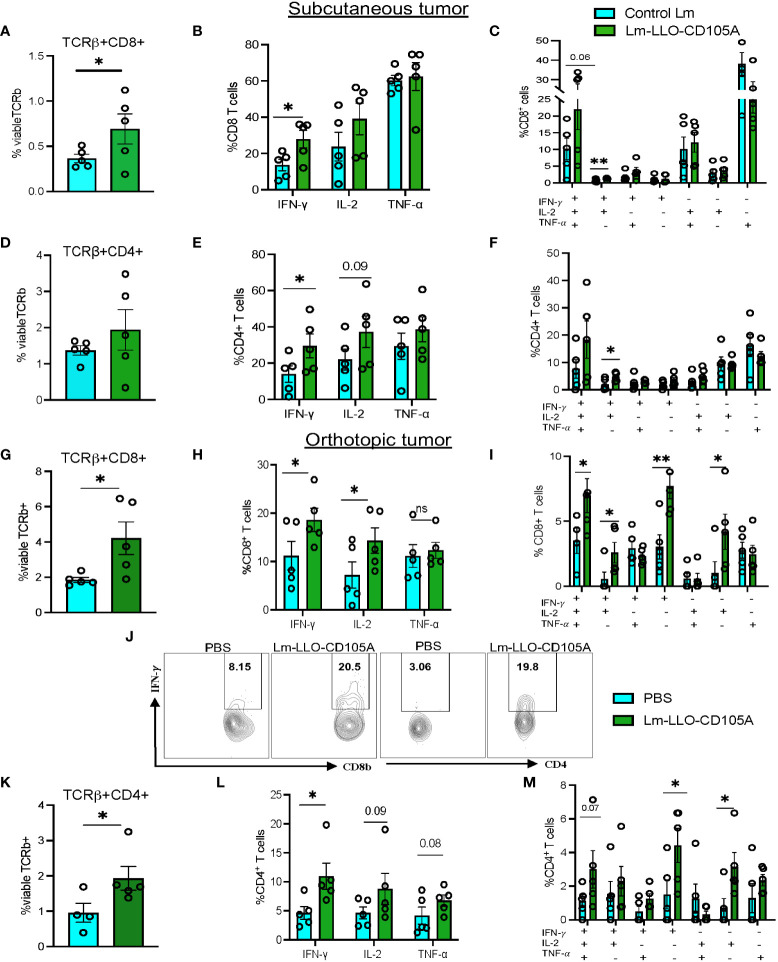
Lm-LLO-CD105A remodels the tumor microenvironment to improve T cell functionality. **(A)** Population of subcutaneous tumor-infiltrating CD8^+^ T cells. **(B)** Cytokine production by CD8^+^ T cells. **(C)** Population of multi-cytokine producing CD8^+^ T cells in the subcutaneous tumor. **(D)** Population of subcutaneous tumor-infiltrating CD4^+^ T cells. **(E)** Cytokine production by CD4^+^ T cells. **(F)** Population of multi-cytokine producing CD4^+^ T cells in the subcutaneous tumor. **(G)** Population of orthotopic tumor-infiltrating CD8^+^ T cells. **(H)** Cytokine production by orthotopic tumor-infiltrating CD8^+^ T cells. **(I)** Population of multi-cytokine producing CD8^+^ T cells in the orthotopic tumor. **(J)** Representative dot plot for IFN-γ production by orthotopic tumor-infiltrating CD8^+^ and CD4^+^ T cells. **(K)**Population of orthotopic tumor-infiltrating CD4^+^ T cells **(L)** Cytokine production by orthotopic tumor-infiltrating CD4^+^ T cells. **(M)**Population of multicytokine producing CD4^+^ T cells in the orthotopic tumor.(n=5/group). Flow cytometry gating strategy for T cell function and myeloid cells is as shown in **Supplementary Figures 3A-D**. Polyfunctional CD4^+,^ and CD8^+^ T cells were identified using the Boolean gating function on flowjo software. Data were analyzed using unpaired t-test. *P < 0.05, **P < 0.01, ns, not significant. All error bars are shown as mean ± SEM.

Modulation of the TME to accommodate immune cell infiltration and enhance anti-tumor immunity is an important aspect of effective tumor immunotherapy (40). To establish the immunomodulatory effect of Lm-LLO-CD105A, we assessed the population of TILs in the TME by flow cytometry. The gating strategy for intracellular cytokine staining is shown in **Supplementary Figure S3A, B**. Lm-LLO-CD105A treated mice demonstrated a marked increase in infiltration of CD8^+^ and CD4^+^ T cells into the TME of subcutaneous tumors (**Figures 4A, D**). Since the functionality of T cells is important for anti-tumor activity, we investigated the cytokine production of both the CD4^+^ and CD8^+^ T cells. In comparison with the Control Lm treatment group in the subcutaneous model, Lm-LLO-CD105A elicited a significantly higher population of IFN-γ producing CD8^+^ and CD4^+^ T cells (**Figures 4B, E**). Further, a significant increase in IFN-γ^+^IL-2^+^ double cytokine production by both tumor-infiltrating CD8^+^and CD4^+^ was obtained in the Lm-LLO-CD105A treated group in comparison to the Control Lm treated group (**Figures 4C, F**).

In the orthotopic tumors, a similar significant increase in infiltrating CD8^+^ and CD4^+^ T cells was observed in the Lm-LLO-CD105A treated group as compared with the group receiving vehicle (PBS) (**Figures 4G, K**). Further, the functionality of these immune cells was significantly elevated, as demonstrated by the increased production of IFN-γ by the CD8^+^ and CD4^+^ T cells in the Lm-LLO-CD105A treated mice (**Figures 4H, J, L**). More importantly, the Lm-LLO-CD105A vaccinated cohort demonstrated a remarkable improvement in the polyfunctionality of CD8^+^T cells as demonstrated by significantly improved triple cytokine-producers (IFN-γ^+^IL-2^+^ TNF-α-^+^) (**Figure 4I**). Further, the tumor-infiltrating CD8^+^ T cells in the Lm-LLO-CD105A treated group showed an effector phenotype as marked by an elevated population of CD8^+^CD62L ^lo^ cells in the orthotopic model (**Supplementary Figure S4C**). Consistent with the observations in the subcutaneous tumors, we found a slight but non-significant improvement in the population of triple cytokine-producing CD4^+^ T cells (**Figure 4M**).

**Figure 5:**
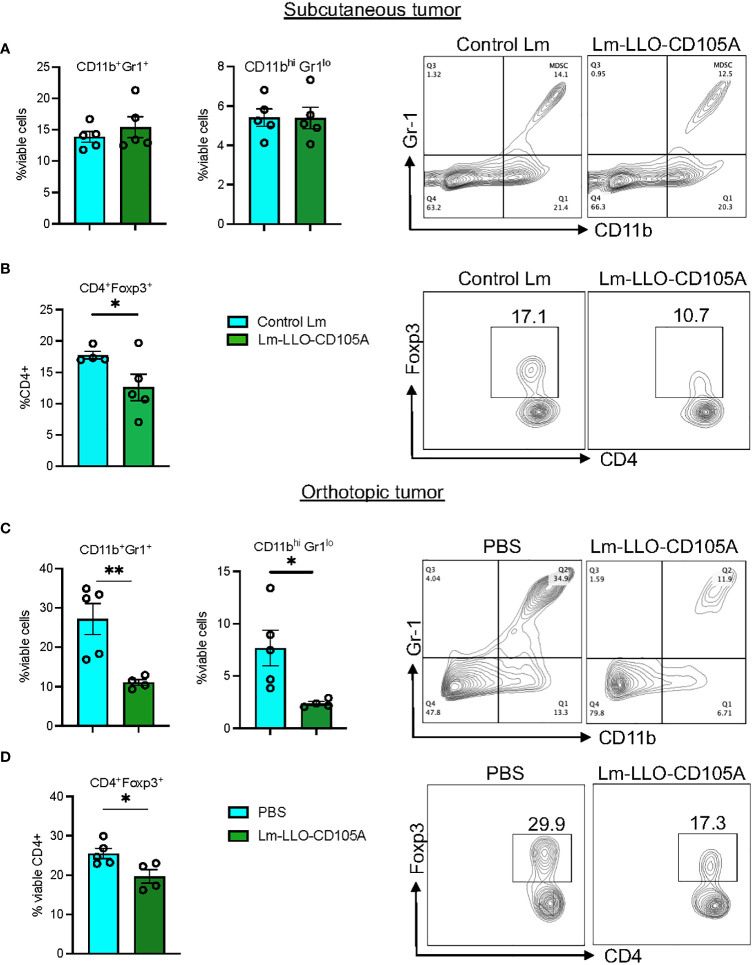
Lm-LLO-CD105 remodels the tumor microenvironment to reduce MDSC and Treg-mediated immunosuppression. **(A)** MDSC population between Control Lm and Lm-LLO-CD105A vaccinated group. **(B)** Statistical representation and dot plot of Treg population in the Lm-LLO-CD105A and control groups. **(C)** MDSC population in the Lm-LLO-CD105A vaccinated group in comparison to the PBS group. **(D)** Flow plot and statistical representation of Treg population in the Lm-LLO-CD105A vaccinated and control groups. Data were analyzed using unpaired t-test. *P < 0.05, **P < 0.01. All error bars are shown as mean ± SEM.

To identify the ability of Lm-LLO-CD105A to reduce the immunosuppressive nature of the TME, we assessed the population of CD4^+^Foxp3^+^ Tregs and MDSCs as shown in **Supplementary Figures S3C, D**. In the subcutaneous model, we did not find a significant difference in the populations of tumor-associated CD11b^+^Gr1^+^ and CD11b^hi^Gr11^lo^ cells between the control groups and the Lm-LLO-CD105A group (**Figure 5A** and **Supplementary Figure S4A**). However, there was a significant reduction in the tumor-associated CD11b^+^Gr1^+^ and CD11b^hi^Gr1^lo^ cell populations in the orthotopic model (**Figure 5C** and **Supplementary Figures S4B, S4D**). In both models, Lm-LLO-CD105A demonstrated an ability to reduce the tumor-associated population of CD4^+^Foxp3^+^ Tregs in comparison with either Control Lm or PBS (**Figures 5B, D**).

### Lm-LLO-CD105A promotes tumor control in a CD8 ^+^ T cell-dependent manner

**Figure 6:**
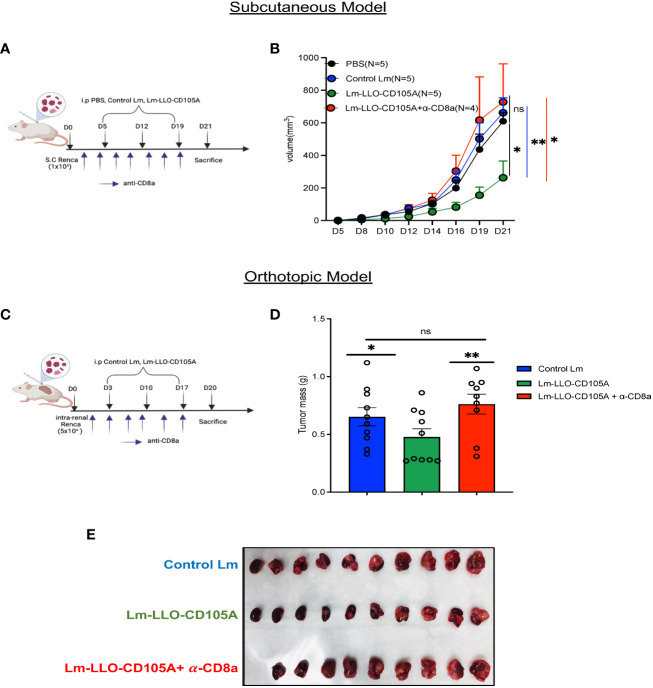
CD8^+^ T cells are responsible for the therapeutic efficacy of Lm-LLO-CD105A. **(A)** Experimental schedule for subcutaneous CD8^+^ depletion studies. **(B)** Longitudinal tumor growth curve. **(C)** Experimental schedule for orthotopic CD8^+^ depletion studies. **(D)** Representative image of diseased kidney. **(E)** Final kidney mass. n=5-10/group. Data were analyzed using unpaired t-test for subcutaneous model **(B)** and Mann-Whitney U test for orthotopic model **(E)**. *P < 0.05, **P < 0.01, ns, not significant. All error bars are shown as mean ± SEM. Vaccination schedule depiction was made in BioRender.

To validate our hypothesis that the anti-tumor efficacy of Lm-LLO-CD105A is related to the significant infiltration and polyfunctionality of cytotoxic CD8^+^ TILs, CD8 lymphocyte depletion experiments were carried out in both the subcutaneous and orthotopic RCC models. In both models, vaccinations and lymphocyte depletion were carried out as depicted (**Figures 6A, C**), and depletion was confirmed in the spleen by flow cytometry (**Supplementary Figures S5A, B**). As expected, the tumor control elicited by the Lm-LLO-CD105A vaccine was lost upon CD8-depletion in both the subcutaneous (**Figure 6B** and **Supplementary Figure S5C**) and orthotopic models (**Figures 6D, E**), indicating that CD8^+^ T cells were the primary drivers of the anti-tumor efficacy of Lm-LLO-CD105A. Further alluding to the importance of CD8^+^T cells, the splenic CD8^+^ T cell population in the Lm-LLO-CD105A vaccinated cohort had enhanced polyfunctionality (**Supplementary Figures S6A-E**), and we observed a significant increase in CD105-specific T cell responses in the spleen of the Lm-LLO-CD105A vaccinated group (**Supplementary Figure S6F**). It has been recently suggested that Lm-based vaccines can also elicit direct tumor cell killing as an additional mechanism of action (41, 42). To evaluate this in our model, we infected Renca cells *in vitro* with either Control Lm or Lm-LLO-CD105A and elucidated the invasive capacity and cytotoxic effect of these vaccines on the tumor cells. While we did observe infection of the Renca cells by both vaccine strains, neither of the Lm-based vaccines demonstrated an appreciable cytotoxic effect. (**Supplementary Figures S1A, B**). Therefore, the anti-tumor efficacy of Lm-LLO-CD105A in this RCC model is likely dependent, in large part, on the induction of an anti-tumor immune response mediated by cytotoxic CD8^+^ T cells.

### The therapeutic effect of Lm-LLO-CD105A is dependent on the high expression of tumoral CD105

**Figure 7:**
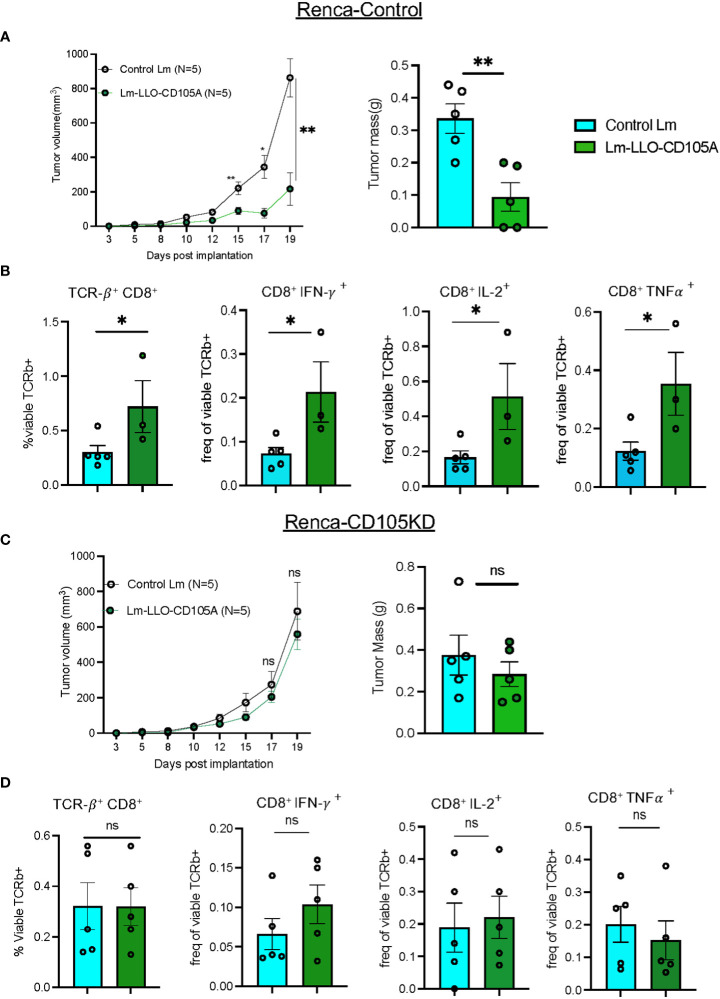
Tumoral CD105 is a robust antigenic target for Lm-LLO-CD015A directed effect. **(A)** Longitudinal tumor growth curve and final tumor mass for Renca-Control tumors. **(B)** CD8^+^ T cell population and cytokine production in Renca-Control group. **(C)** Longitudinal tumor growth curve and final tumor mass for Renca-CD105KD. **(D)** CD8^+^ T cell population and cytokine production in Renca-CD105KD group. (n=5/group). Data were analyzed using unpaired t-test. *P < 0.05, **P < 0.01. All error bars are shown as mean ± SEM. ns, not significant.

Previous studies demonstrated that Lm-LLO-CD105A likely impedes tumor growth by its ability to target tumor vasculature-associated CD105 expression in a murine model of breast cancer in which the tumor cells themselves do not express CD105 (19, 43). However, in RCC, the tumor cells directly express CD105 and a recent study found that high CD105 expression by RCC tumor cells is a better predictor of poor prognosis than high tumor-associated vascular endothelial cell expression of CD105 (15). Therefore, to determine if the efficacy of Lm-LLO-CD105A is dependent on high tumoral CD105 expression, we generated a Renca cell line that is deficient in CD105 (Renca-CD105KD) and confirmed by western blot (**Supplementary Figure S7A**). Subsequently, mice were implanted s.c with either the Renca-CD105KD or the control cell line (Renca-Control) and treated with Control Lm or Lm-LLO-CD105A. As expected, the Lm-LLO-CD105A vaccine effectively controlled tumor growth in the Renca-Control group (**Figure 7A** and **Supplementary Figure S7B**). Interestingly, there was no therapeutic efficacy observed after Lm-LLO-CD105A treatment in the mice bearing Renca-CD105KD (**Figure 7C** and **Supplementary Figure S7C**). While there was a significant increase in the tumor-infiltrating CD8^+^ T cells in the mice bearing Renca-Control tumors after Lm-LLO-CD105A treatment as expected (**Figure 7B**), CTL infiltration was not increased in mice bearing Renca-CD105KD tumors (**Figure 7D**). Further, there was a significant increase in cytokine-producing CD8^+^ T cells in the Renca-Control tumor-bearing mice after treatment with Lm-LLO-CD105A, but there was no similar improvement in cytokine production by the CD8^+^T cells in the mice bearing Renca-CD105KD tumors. (**Figures 7B, D**). Overall, this data suggests that the anti-tumor efficacy of Lm-LLO-CD105A is dependent on the expression of CD105 by the tumor cells.

### Lm-LLO-CD105A does not impact the wound healing and health of mice

**Figure 8:**
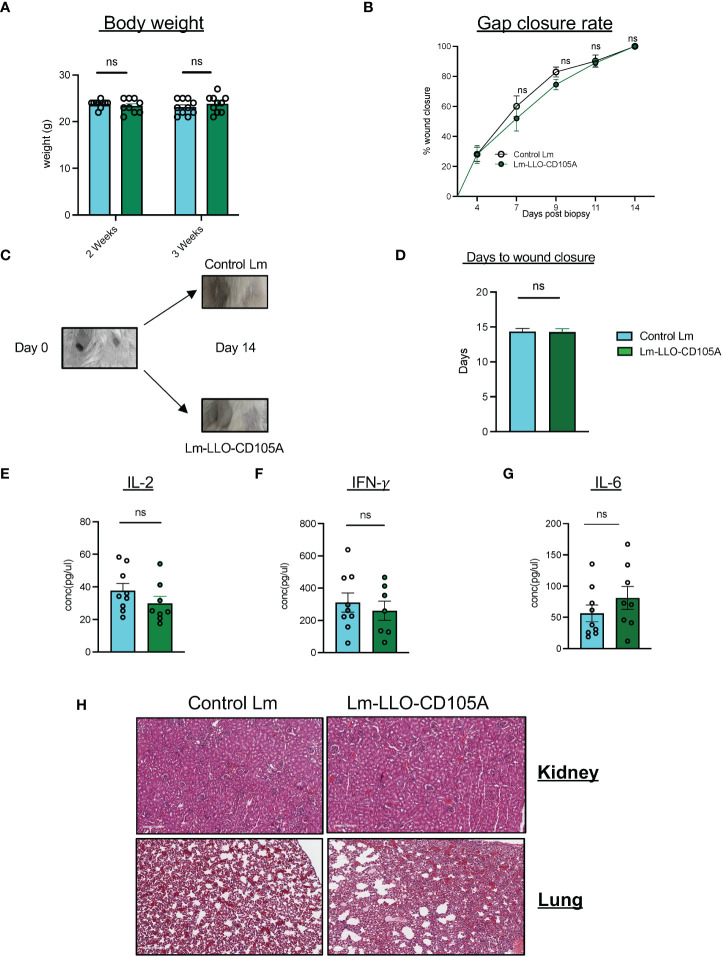
Lm-LLO-CD105 has a high safety profile with no effect on wound healing. **(A)** Body weight of experimental mice post-vaccination. **(B)** Rate of wound closure after skin biopsy punch between either vaccination groups. **(C)** Representative image of biopsy punches before and after wound closure. **(D)** Days to wound closure after biopsy punch. **(E, F)** Serum concentration of proinflammatory cytokines including, **(E)** IL-2. **(F)** IFN-γ, and **(G)** IL-6 between Control Lm and Lm-LLO-CD105A groups. **(H)** Representative images of the immune infiltrates to the healthy kidney and lung tissues between the Control Lm and Lm-LLO-CD105A vaccinated groups. n=9-10/group. Data were analyzed using unpaired t-test. *P < 0.05, ns, not significant. All error bars are shown as mean ± SEM.

Since CD105 is involved in normal physiological processes, specifically wound healing (44), the safety profile of Lm-LLO-CD105A administration was evaluated in non-tumor-bearing mice. There was no observable difference in the overall health of the mice immunized with either Lm-LLO-CD105A or Control Lm as determined by monitoring the weight of the animals (**Figure 8A**). Three weeks after the last vaccine dose, we also performed a wound healing assay as previously described (31, 45). Immunization with Lm-LLO-CD105A did not affect the rate of wound closure (**Figure 8B**). Similarly, we did not observe a difference in the time to wound closure between the Lm-LLO-CD105A and Control Lm treatment groups (**Figures 8C, D**). To determine if Lm-LLO-CD105A treatment elicited systemic inflammation that may be detrimental to the health of experimental mice, serum was collected after complete gap closure and subjected to cytokine ELISA for IFNγ, IL-2, and IL-6. There was no significant difference in the basal serum level of these cytokines between the Lm-LLO-CD105A and Control Lm vaccination groups. (**Figures 8E-G**).

To ensure that Lm-LLO-CD105A vaccine does not cause off-target effects related to the expression of CD105 on normal tissues, healthy kidney tissues from vaccinated and non-tumor bearing mice were subjected to H&E staining and the population of infiltrating lymphocytes was quantified (**Figure 8H**). There was no significant difference in the infiltration of lymphocytes into the healthy kidney of the Control Lm and Lm-LLO-CD105A treatment groups. The culmination of this data suggests that treatment with Lm-LLO-CD105A is generally safe and well tolerated.

## Discussion

The scientific rationale for therapeutic targeting of CD105 is partly due to its ubiquitous expression on tumor vasculature-associated endothelial cells (46, 47). Based on this, previous work by us and others have demonstrated the anti-tumor efficacy of targeting tumor vasculature-associated CD105 (18, 38, 43, 48). Herein, we show the safety and efficacy of targeting tumor vasculature-associated endothelial and tumor cell-expressed CD105 by an Lm-based vaccine, Lm-LLO-CD105A, in both subcutaneous and orthotopic models of RCC.

Remodeling of the TME to reduce its immunosuppressive phenotype is essential to achieve optimum therapeutic benefit from tumor immunotherapies (40). Previous studies that utilized Lm-based vaccines demonstrated the ability to control tumor burden correlated with enhanced CTL infiltration and a reduction in immunosuppressive cell populations in the TME (27). We confirmed this anti-tumor mechanism by showing that Lm-LLO-CD105A significantly increased infiltration and function of TILs and reduced the population of tumor-associated Tregs and MDSCs (Figs. 4 and 5). Indeed, efficient control of MDSCs is characteristic of Lm-based vaccines as they can directly infect MDSCs, leading to their death and/or reverting them into a less suppressive phenotype (28). Nevertheless, the anti-tumor mechanism of Lm-LLO-CD105A strongly relied on CD8^+^ T cells as the anti-tumor efficacy of Lm-LLO-CD105A was lost upon antibody-mediated depletion of CD8^+^ T cells.

Notably, while the efficacy of Lm-LLO-CD105A was significant in both subcutaneous and orthotopic models of murine RCC (**Figure 2**), this therapeutic strategy did not result in complete tumor regression in all mice. However, we did observe an increase in the expression of PD-1 on the CD8^+^ TIL and significantly increased mRNA expression of *Pd-l1* in the tumor tissues from the Lm-LLO-CD105A treated mice (**Supplementary Figures S4E, F**), suggesting that the combination of Lm-LLO-CD105A with anti-PD-1/PD-L1 therapy may provide synergistic therapeutic efficacy. The combination of anti-PD-1 with cancer vaccines has proven to be beneficial in preclinical and clinical trials (49). Furthermore, Lm-based vaccines have been successfully combined with anti-PD-1 to improve therapeutic anti-tumor efficacy (25).

Aside from the anti-tumoral effect of Lm-LLO-CD105A, we demonstrated a significant anti-angiogenic effect, owing to a reduction in CD31 and VEGFR2 expression within the TME (**Figure 3**). CD105-directed approaches for the treatment of RCC have previously been explored in several clinical trials that utilized the anti-CD105 monoclonal antibody,TRC105 (21, 22).However, this passive antibody-mediated approach has, so far, offered no improved benefit over the current standards of care (21, 22, 50). As such, we envision that an active CTL-based approach, such as that afforded by Lm-LLO-CD105A, will provide improved efficacy and significant benefit to RCC patients. A challenge often encountered after anti-angiogenic therapy is that the integrity of the tumor vasculature is compromised, and it becomes difficult for additional therapy and immune cells to infiltrate the tumor (45, 51). However, as evidenced by our results, it appears that the disruption in blood vessels mediated by Lm-LLO-CD105A is insufficient to prevent the trafficking of immune cells to the tumor.

The Renca model utilized in the study represents the expression of CD105 on both the tumor cell and the tumor-associated vasculature (**Figures 1C-E**). The loss of therapeutic efficacy of Lm-LLO-CD105A upon downregulation of CD105 in Renca tumor cells (**Figure 7C**) was unexpected as we had seen a significant reduction in tumor-associated vasculature. However, prior work showed that the efficacy of an anti-angiogenic Lm-based vaccine targeting the vascular antigen VEGFR2 was dependent on epitope spreading to tumor-associated antigens as responses to tumor vasculature alone were insufficient (45).Importantly, Saroufim et al. have recently shown that high tumoral expression of CD105 is more predictive of poor prognosis than high endothelial expression in RCC (15).

Interestingly, in RCC, CD105 demarcates a subpopulation of tumor cells that are characterized as tumor-initiating cells with stem cell properties (36). As such, it is possible that Lm-LLO-CD105A’s anti-tumor efficacy is primarily mediated by eradicating this highly malignant stem cell population. In summary, our data suggest that patients in advanced stages of RCC with high tumoral CD105 expression could potentially benefit from a CD105-targeted therapy.

As with other TAA-targeting therapies, there is a concern regarding the off-target immune effect in otherwise healthy tissues (52). This is of less concern as various studies that exploited CD105 for therapeutic applications reported little to no adverse effects (38, 48). Similarly, the monoclonal antibody TRC105 was well-tolerated in monotherapy and combination therapy in clinical trials (53, 54). Here, we confirmed that this CTL-based therapy against CD105 did not impact the wound-healing capacities of the mice, nor did it affect the overall health of experimental mice (**Figure 8**).

As demonstrated in this manuscript, Lm-based vaccine directed against tumor vasculature-associated and tumoral CD105 constrained the growth of tumors by activating cytotoxic tumor-specific immunity. The benefit of this strategy is underscored by the resistance that often occurs to agents that target only the tumor-associated endothelium in RCC (55, 56). Targeting CD105 with an Lm-based vaccine in RCC potentially subverts this limitation, as we demonstrated that the antitumor effect of Lm-LLO-CD105A resulted in both endothelial and tumor cell targeting. While our study comprehensively elucidated the anti-tumor efficacy of Lm-LLO-CD105A, its ability to improve overall survival in RCC in comparison to currently approved standard-of-care remains unknown. As such, further work is required to elucidate the effect on survival in comparison with currently approved therapies such as immune checkpoint inhibitors and tyrosine kinase inhibitors (2, 57). Despite demonstrating robust anti-tumor efficacy in our studies, murine models do not perfectly represent the human RCC (58, 59). Nevertheless, Lm-based vaccines that demonstrated efficacy in preclinical trials have advanced to clinical trials and demonstrated promising results in humans (23). As such, we propose that targeting CD105 with an Lm-based vaccine may be a promising strategy for improving survival in patients with RCC.

## Data availability statement

The original contributions presented in the study are included in the article/**Supplementary Material**. Further inquiries can be directed to the corresponding author.

## Ethics statement

The animal study was reviewed and approved by Institutional Animal Care and Use Committee (IACUC) of Texas Tech University Health Sciences Center.

## Author contributions

The study was designed and conceptualized by LMW. MO performed experiments, analyzed data, interpreted results, prepared figures, and drafted the manuscript. HMN provided support in orthotopic experiments. AS contributed to immunofluorescence (IF) studies and quantification, BR contributed to the assembly of the CRISPR-Cas9 vector and IF studies, WP provided support during *in vitro* cytotoxicity and infectivity assays, MMM and LMW provided supervision. LMW acquired funding, contributed to result interpretation, and edited the manuscript. All authors contributed to the article and approved the submitted version.

## Funding

This work is supported by the (NIH) 1R15CA216205-01 grant to Laurence Wood.

## Acknowledgments

We thank Dr. Devin Lowe at the Department of Immunotherapeutics and Biotechnology, TTUHSC, for providing us with the CRISPR-Cas9 vector.

## Conflict of interest

The authors declare that the research was conducted in the absence of any commercial or financial relationships that could be construed as a potential conflict of interest.

## Publisher’s note

All claims expressed in this article are solely those of the authors and do not necessarily represent those of their affiliated organizations, or those of the publisher, the editors and the reviewers. Any product that may be evaluated in this article, or claim that may be made by its manufacturer, is not guaranteed or endorsed by the publisher.

## References

[1] Padala SA Barsouk A Thandra KC Saginala K Mohammed A Vakiti A. Epidemiology of renal cell carcinoma. World J Oncol (2020) 11(3):79–87. doi: 10.14740/wjon1279 32494314 PMC7239575

[2] Tenold M Ravi P Kumar M Bowman A Hammers H Choueiri TK. Current approaches to the treatment of advanced or metastatic renal cell carcinoma. Am Soc Clin Oncol Educ Book (2020) 40:1–10. doi: 10.1200/EDBK_279881 32239988

[3] Makhov P Joshi S Ghatalia P Kutikov A Uzzo RG Kolenko VM. Resistance to systemic therapies in clear cell renal cell carcinoma: Mechanisms and management strategies. Mol Cancer Ther (2018) 17(7):1355–64. doi: 10.1158/1535-7163.MCT-17-1299 PMC6034114 29967214

[4] Motzer RJ Escudier B Tomczak P Hutson TE Michaelson MD Negrier S. Axitinib versus sorafenib as second-line treatment for advanced renal cell carcinoma: overall survival analysis and updated results from a randomised phase 3 trial. Lancet Oncol (2013) 14(6):552–62. doi: 10.1016/S1470-2045(13)70093-7 23598172

[5] Motzer RJ Hutson TE Cella D Reeves J Hawkins R Guo J. Pazopanib versus sunitinib in metastatic renal-cell carcinoma. N Engl J Med (2013) 369(8):722–31. doi: 10.1056/NEJMoa1303989 23964934

[6] Lee CH Hotker AM Voss MH Feldman DR Woo KM Patil S. Bevacizumab monotherapy as salvage therapy for advanced clear cell renal cell carcinoma pretreated with targeted drugs. Clin Genitourin Cancer (2016) 14(1):56–62. doi: 10.1016/j.clgc.2015.07.010 26404107 PMC4965701

[7] Choueiri TK Albiges L Atkins MB Bakouny Z Bratslavsky G Braun DA. From basic science to clinical translation in kidney cancer: A report from the second kidney cancer research summit. Clin Cancer Res (2022) 28(5):831–9. doi: 10.1158/1078-0432.CCR-21-3238 PMC9223120 34965942

[8] Buczek M Escudier B Bartnik E Szczylik C Czarnecka A. Resistance to tyrosine kinase inhibitors in clear cell renal cell carcinoma: From the patient’s bed to molecular mechanisms. Biochim Biophys Acta (2014) 1845(1):31–41. doi: 10.1016/j.bbcan.2013.10.001 24135488

[9] Itatani Y Kawada K Yamamoto T Sakai Y. Resistance to anti-angiogenic therapy in cancer-alterations to anti-VEGF pathway. Int J Mol Sci (2018) 19(4):1232. doi: 10.3390/ijms19041232 29670046 PMC5979390

[10] Haibe Y Kreidieh M El Hajj H Khalifeh I Mukherji D Temraz S. Resistance mechanisms to anti-angiogenic therapies in cancer. Front Oncol (2020) 10:221. doi: 10.3389/fonc.2020.00221 32175278 PMC7056882

[11] Ferrari G Cook BD Terushkin V Pintucci G Mignatti P. Transforming growth factor-beta 1 (TGF-beta1) induces angiogenesis through vascular endothelial growth factor (VEGF)-mediated apoptosis. J Cell Physiol (2009) 219(2):449–58. doi: 10.1002/jcp.21706 PMC2749291 19180561

[12] Goumans MJ Liu Z ten Dijke P. TGF-beta signaling in vascular biology and dysfunction. Cell Res (2009) 19(1):116–27. doi: 10.1038/cr.2008.326 19114994

[13] Kumar P Wang JM Bernabeu C. CD 105 and angiogenesis. J Pathol (1996) 178(4):363–6. doi: 10.1002/(SICI)1096-9896(199604)178:4<363::AID-PATH491>3.0.CO;2-8 8691311

[14] Warrington K Hillarby MC Li C Letarte M Kumar S. Functional role of CD105 in TGF-beta1 signalling in murine and human endothelial cells. Anticancer Res (2005) 25(3B):1851–64. 16158917

[15] Saroufim A Messai Y Hasmim M Rioux N Iacovelli R Verhoest G. Tumoral CD105 is a novel independent prognostic marker for prognosis in clear-cell renal cell carcinoma. Br J Cancer (2014) 110(7):1778–84. doi: 10.1038/bjc.2014.71 PMC3974088 24594997

[16] Hu J Guan W Liu P Dai J Tang K Xiao H. Endoglin is essential for the maintenance of self-renewal and chemoresistance in renal cancer stem cells. Stem Cell Rep (2017) 9(2):464–77. doi: 10.1016/j.stemcr.2017.07.009 PMC5550272 28793246

[17] Saeednejad Zanjani L Madjd Z Abolhasani M Shariftabrizi A Rasti A Asgari M. Expression of CD105 cancer stem cell marker in three subtypes of renal cell carcinoma. Cancer biomark (2018) 21(4):821–37. doi: 10.3233/CBM-170755 PMC13078323 29286924

[18] Lee SH Mizutani N Mizutani M Luo Y Zhou H Kaplan C. Endoglin (CD105) is a target for an oral DNA vaccine against breast cancer. Cancer Immunol Immunother (2006) 55(12):1565–74. doi: 10.1007/s00262-006-0155-5 PMC11030801 16565828

[19] Wood LM Pan ZK Guirnalda P Tsai P Seavey M Paterson Y. Targeting tumor vasculature with novel listeria-based vaccines directed against CD105. Cancer Immunol Immunother (2011) 60(7):931–42. doi: 10.1007/s00262-011-1002-x PMC4438988 21431419

[20] Tsujie M Uneda S Tsai H Seon BK. Effective anti-angiogenic therapy of established tumors in mice by naked anti-human endoglin (CD105) antibody: Differences in growth rate and therapeutic response between tumors growing at different sites. Int J Oncol (2006) 29(5):1087–94. doi: 10.3892/ijo.29.5.1087 17016638

[21] Choueiri TK Zakharia Y Pal S Kocsis J Pachynski R Poprach A. Clinical results and biomarker analyses of axitinib and TRC105 versus axitinib alone in patients with advanced or metastatic renal cell carcinoma (TRAXAR). Oncologist (2021) 26(7):560–e1103. doi: 10.1002/onco.13777 33829609 PMC8265348

[22] Dorff TB Longmate JA Pal SK Stadler WM Fishman MN Vaishampayan UN. Bevacizumab alone or in combination with TRC105 for patients with refractory metastatic renal cell cancer. Cancer (2017) 123(23):4566–73. doi: 10.1002/cncr.30942 PMC5726867 28832978

[23] Oladejo M Paterson Y Wood LM. Clinical experience and recent advances in the development of listeria-based tumor immunotherapies. Front Immunol (2021) 12:642316. doi: 10.3389/fimmu.2021.642316 33936058 PMC8081050

[24] Wood LM Paterson Y. Attenuated listeria monocytogenes: a powerful and versatile vector for the future of tumor immunotherapy. Front Cell Infect Microbiol (2014) 4:51. doi: 10.3389/fcimb.2014.00051 24860789 PMC4026700

[25] Mkrtichyan M Chong N Abu Eid R Wallecha A Singh R Rothman J. Anti-PD-1 antibody significantly increases therapeutic efficacy of listeria monocytogenes (Lm)-LLO immunotherapy. J Immunother Cancer (2013) 1:15. doi: 10.1186/2051-1426-1-15 24829751 PMC4019896

[26] Morrow ZT Powers ZM Sauer JD. Listeria monocytogenes cancer vaccines: bridging innate and adaptive immunity. Curr Clin Microbiol Rep (2019) 6(4):213–24. doi: 10.1007/s40588-019-00133-4 PMC7560980 33072493

[27] Wallecha A Singh R Malinina I. Listeria monocytogenes (Lm)-LLO immunotherapies reduce the immunosuppressive activity of myeloid-derived suppressor cells and regulatory T cells in the tumor microenvironment. J Immunother (2013) 36(9):468–76. doi: 10.1097/CJI.0000000000000000 24145358

[28] Chandra D Jahangir A Quispe-Tintaya W Einstein MH Gravekamp C. Myeloid-derived suppressor cells have a central role in attenuated listeria monocytogenes-based immunotherapy against metastatic breast cancer in young and old mice. Br J Cancer (2013) 108(11):2281–90. doi: 10.1038/bjc.2013.206 PMC3681012 23640395

[29] Faustino-Rocha A Oliveira PA Pinho-Oliveira J Teixeira-Guedes C Soares-Maia R da Costa RG. Estimation of rat mammary tumor volume using caliper and ultrasonography measurements. Lab Anim (NY) (2013) 42(6):217–24. doi: 10.1038/laban.254 23689461

[30] Murphy KA James BR Wilber A Griffith TS. A syngeneic mouse model of metastatic renal cell carcinoma for quantitative and longitudinal assessment of preclinical therapies. J Vis Exp (2017) 122):e55080. doi: 10.3791/55080 PMC5564692 28448047

[31] Niethammer AG Xiang R Becker JC Wodrich H Pertl U Karsten G. A DNA vaccine against VEGF receptor 2 prevents effective angiogenesis and inhibits tumor growth. Nat Med (2002) 8(12):1369–75. doi: 10.1038/nm1202-794 12415261

[32] Feldman AT Wolfe D. Tissue processing and hematoxylin and eosin staining. Methods Mol Biol (2014) 1180:31–43. doi: 10.1007/978-1-4939-1050-2_3 25015141

[33] Nomura Y Yamashita T Oishi N Nio K Hayashi T Yoshida M. *De novo* emergence of mesenchymal stem-like CD105(+) cancer cells by cytotoxic agents in human hepatocellular carcinoma. Transl Oncol (2017) 10(2):184–9. doi: 10.1016/j.tranon.2017.01.005 PMC5299205 28182993

[34] Zhang J Sang X Zhang R Chi J Bai W. CD105 expression is associated with invasive capacity in ovarian cancer and promotes invasiveness by inhibiting NDRG1 and regulating the epithelial-mesenchymal transition. Am J Transl Res (2021) 13(11):12461–79. PMC8661161 34956466

[35] Fujiwara K Ohuchida K Ohtsuka T Mizumoto K Shindo K Ikenaga N. Migratory activity of CD105+ pancreatic cancer cells is strongly enhanced by pancreatic stellate cells. Pancreas (2013) 42(8):1283–90. doi: 10.1097/MPA.0b013e318293e7bd 24308064

[36] Bussolati B Bruno S Grange C Ferrando U Camussi G. Identification of a tumor-initiating stem cell population in human renal carcinomas. FASEB J (2008) 22(10):3696–705. doi: 10.1096/fj.08-102590 18614581

[37] Sobczuk P Brodziak A Khan MI Chhabra S Fiedorowicz M Welniak-Kaminska M. Choosing the right animal model for renal cancer research. Transl Oncol (2020) 13(3):100745. doi: 10.1016/j.tranon.2020.100745 32092671 PMC7036425

[38] Jarosz M Jazowiecka-Rakus J Cichon T Glowala-Kosinska M Smolarczyk R Smagur A. Therapeutic antitumor potential of endoglin-based DNA vaccine combined with immunomodulatory agents. Gene Ther (2013) 20(3):262–73. doi: 10.1038/gt.2012.28 22495576

[39] Albelda SM Muller WA Buck CA Newman PJ. Molecular and cellular properties of PECAM-1 (endoCAM/CD31): A novel vascular cell-cell adhesion molecule. J Cell Biol (1991) 114(5):1059–68. doi: 10.1083/jcb.114.5.1059 PMC2289123 1874786

[40] Locy H de Mey S de Mey W De Ridder M Thielemans K Maenhout SK. Immunomodulation of the tumor microenvironment: Turn foe into friend. Front Immunol (2018) 9:2909. doi: 10.3389/fimmu.2018.02909 30619273 PMC6297829

[41] Kim SH Castro F Paterson Y Gravekamp C. High efficacy of a listeria-based vaccine against metastatic breast cancer reveals a dual mode of action. Cancer Res (2009) 69(14):5860–6. doi: 10.1158/0008-5472.CAN-08-4855 PMC3127451 19584282

[42] Selvanesan BC Chandra D Quispe-Tintaya W Jahangir A Patel A Meena K. Listeria delivers tetanus toxoid protein to pancreatic tumors and induces cancer cell death in mice. Sci Transl Med (2022) 14(637):eabc1600. doi: 10.1126/scitranslmed.abc1600 35320003 PMC9031812

[43] Ghouse SM Vadrevu SK Manne S Reese B Patel J Patel B. Therapeutic targeting of vasculature in the premetastatic and metastatic niches reduces lung metastasis. J Immunol (2020) 204(4):990–1000. doi: 10.4049/jimmunol.1901208 31900334 PMC7012400

[44] Valluru M Staton CA Reed MW Brown NJ. Transforming growth factor-beta and endoglin signaling orchestrate wound healing. Front Physiol (2011) 2:89. doi: 10.3389/fphys.2011.00089 22164144 PMC3230065

[45] Seavey MM Maciag PC Al-Rawi N Sewell D Paterson Y. An anti-vascular endothelial growth factor receptor 2/fetal liver kinase-1 listeria monocytogenes anti-angiogenesis cancer vaccine for the treatment of primary and metastatic her-2/neu+ breast tumors in a mouse model. J Immunol (2009) 182(9):5537–46. doi: 10.4049/jimmunol.0803742 PMC2850569 19380802

[46] Fonsatti E Altomonte M Nicotra MR Natali PG Maio M. Endoglin (CD105): A powerful therapeutic target on tumor-associated angiogenetic blood vessels. Oncogene (2003) 22(42):6557–63. doi: 10.1038/sj.onc.1206813 14528280

[47] Dallas NA Samuel S Xia L Fan F Gray MJ Lim SJ. Endoglin (CD105): a marker of tumor vasculature and potential target for therapy. Clin Cancer Res (2008) 14(7):1931–7. doi: 10.1158/1078-0432.CCR-07-4478 18381930

[48] Mo F Duan S Jiang X Yang X Hou X Shi W. Nanobody-based chimeric antigen receptor T cells designed by CRISPR/Cas9 technology for solid tumor immunotherapy. Signal Transduct Target Ther (2021) 6(1):80. doi: 10.1038/s41392-021-00462-1 33627635 PMC7904846

[49] Zhao J Chen Y Ding ZY Liu JY. Safety and efficacy of therapeutic cancer vaccines alone or in combination with immune checkpoint inhibitors in cancer treatment. Front Pharmacol (2019) 10:1184. doi: 10.3389/fphar.2019.01184 31680963 PMC6798079

[50] Choueiri TK Michaelson MD Posadas EM Sonpavde GP McDermott DF Nixon AB. An open label phase ib dose escalation study of TRC105 (Anti-endoglin antibody) with axitinib in patients with metastatic renal cell carcinoma. Oncologist (2019) 24(2):202–10. doi: 10.1634/theoncologist.2018-0299 PMC6369938 30190302

[51] Van der Veldt AA Lubberink M Bahce I Walraven M de Boer MP Greuter HN. Rapid decrease in delivery of chemotherapy to tumors after anti-VEGF therapy: implications for scheduling of anti-angiogenic drugs. Cancer Cell (2012) 21(1):82–91. doi: 10.1016/j.ccr.2011.11.023 22264790

[52] Li L Goedegebuure SP Gillanders W. Cancer vaccines: shared tumor antigens return to the spotlight. Signal Transduct Target Ther (2020) 5(1):251. doi: 10.1038/s41392-020-00364-8 33127890 PMC7599325

[53] Rosen LS Hurwitz HI Wong MK Goldman J Mendelson DS Figg WD. A phase I first-in-human study of TRC105 (Anti-endoglin antibody) in patients with advanced cancer. Clin Cancer Res (2012) 18(17):4820–9. doi: 10.1158/1078-0432.CCR-12-0098 PMC3432706 22767667

[54] Jones RL Ravi V Brohl AS Chawla S Ganjoo KN Italiano A. Efficacy and safety of TRC105 plus pazopanib vs pazopanib alone for treatment of patients with advanced angiosarcoma: A randomized clinical trial. JAMA Oncol (2022) 8(5):740–7. doi: 10.1001/jamaoncol.2021.3547 PMC8972152 35357396

[55] Jimenez-Valerio G Martinez-Lozano M Bassani N Vidal A Ochoa-de-Olza M Suarez C. Resistance to antiangiogenic therapies by metabolic symbiosis in renal cell carcinoma PDX models and patients. Cell Rep (2016) 15(6):1134–43. doi: 10.1016/j.celrep.2016.04.015 PMC4870515 27134180

[56] Joosten SC Hamming L Soetekouw PM Aarts MJ Veeck J van Engeland M. Resistance to sunitinib in renal cell carcinoma: From molecular mechanisms to predictive markers and future perspectives. Biochim Biophys Acta (2015) 1855(1):1–16. doi: 10.1016/j.bbcan.2014.11.002 25446042

[57] Bosma NA Warkentin MT Gan CL Karim S Heng DYC Brenner DR. Efficacy and safety of first-line systemic therapy for metastatic renal cell carcinoma: A systematic review and network meta-analysis. Eur Urol Open Sci (2022) 37:14–26. doi: 10.1016/j.euros.2021.12.007 35128482 PMC8792068

[58] Schokrpur S Hu J Moughon DL Liu P Lin LC Hermann K. CRISPR-mediated VHL knockout generates an improved model for metastatic renal cell carcinoma. Sci Rep (2016) 6:29032. doi: 10.1038/srep29032 27358011 PMC4928183

[59] Wolf MM Kimryn Rathmell W Beckermann KE. Modeling clear cell renal cell carcinoma and therapeutic implications. Oncogene (2020) 39(17):3413–26. doi: 10.1038/s41388-020-1234-3 PMC7194123 32123314

