# Supplementary material for: Listeria-based immunotherapy directed against CD105 exerts anti-angiogenic and anti-tumor efficacy in renal cell carcinoma
Source: Front Immunol. 2022 Nov 11;13:1038807. doi: 10.3389/fimmu.2022.1038807 (PMC9692019; doi:10.3389/fimmu.2022.1038807)
Supplement: Supplementary file 1 [file DataSheet_1.pdf]

Supplementary Figures

Supplementary Figure S1

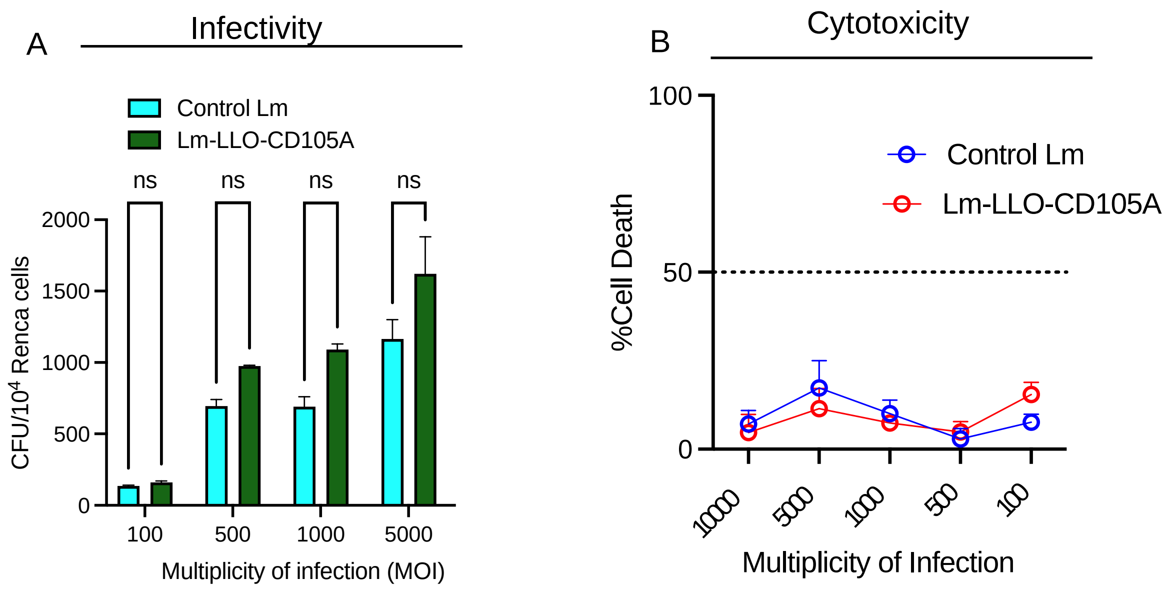

Supplementary Figure S1. Lm-based vaccine does not induce direct tumor cell killing. Renca cells were cultured with varying MOIs of Control Lm or Lm-LLO-CD105A, and infection or cytotoxicity was determined **(A)** Infectivity rate, expressed as CFU/10,000 cells in Renca **(B)** Dose-response curve to Lm-based vaccines in Renca cells

Supplementary Figure S2

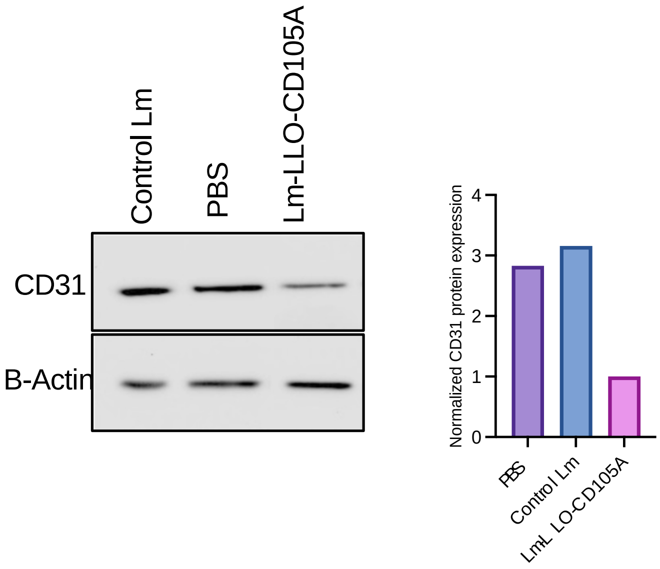

Supplementary Figure S2. Protein expression of CD31, a marker of angiogenesis after therapy with Lm-based vaccines.

Supplementary Figure S3

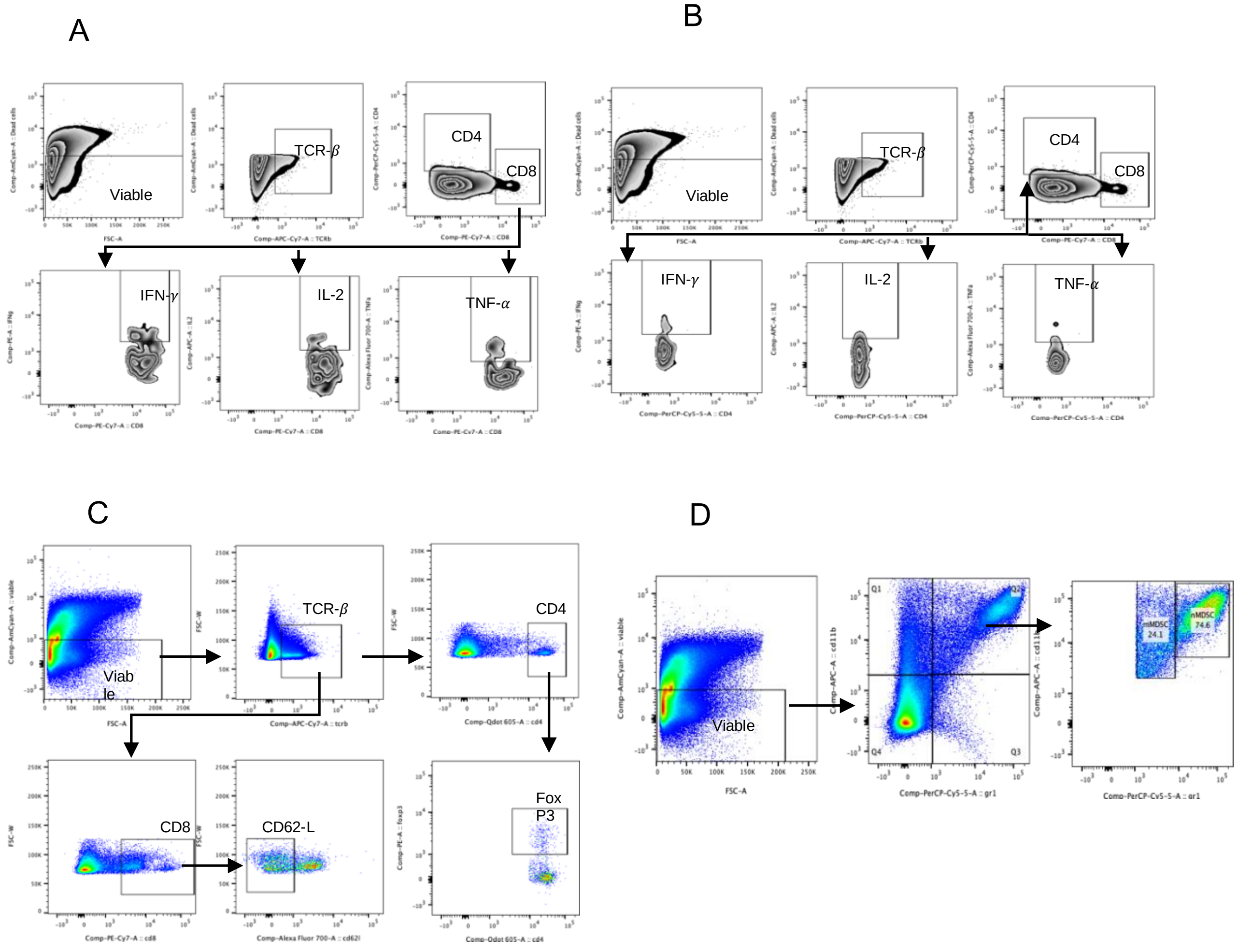

Supplementary Figure S3. Gating strategy for tumor-infiltrating lymphocytes and myeloid cells. (A-D) Following stimulation and staining, viable cells were discriminated with Amcyan gating followed by sub-gating with TCR-b. TCR-b+ cells were further sub-gated to CD4 and CD8+ T cells. **(A)**IFNg, IL2, and TNF-a were then sub-gated on CD8+ T cells or **(B)** CD4+ T cells. The combination/Boolean gating function on Flowjo software was used to identify the multicytokine-producing population **(C)** Dead cells were excluded using Amcyan staining followed by TCR-b selection, CD4 and $CD8^+$ T cells were then gated on TCRb+ cells. On $CD8^+$ cells, the population of $CD62L^{lo}$ cells was gated, and on $CD4^+$ cells population of $Foxp3^+$ cells were gated **(D)** Viable cells were discriminated with Amcyan staining, followed by double gating against CD11b and Gr1 to select $CD11b^+Gr1^+$ cells. $CD11b^+Gr1^+$ cells were further sub gated to characterize $CD11b^{hi}Gr1^{hi}$ and $CD11b^{hi}$ $Gr1^{lo}$ cells

Supplementary Figure S4

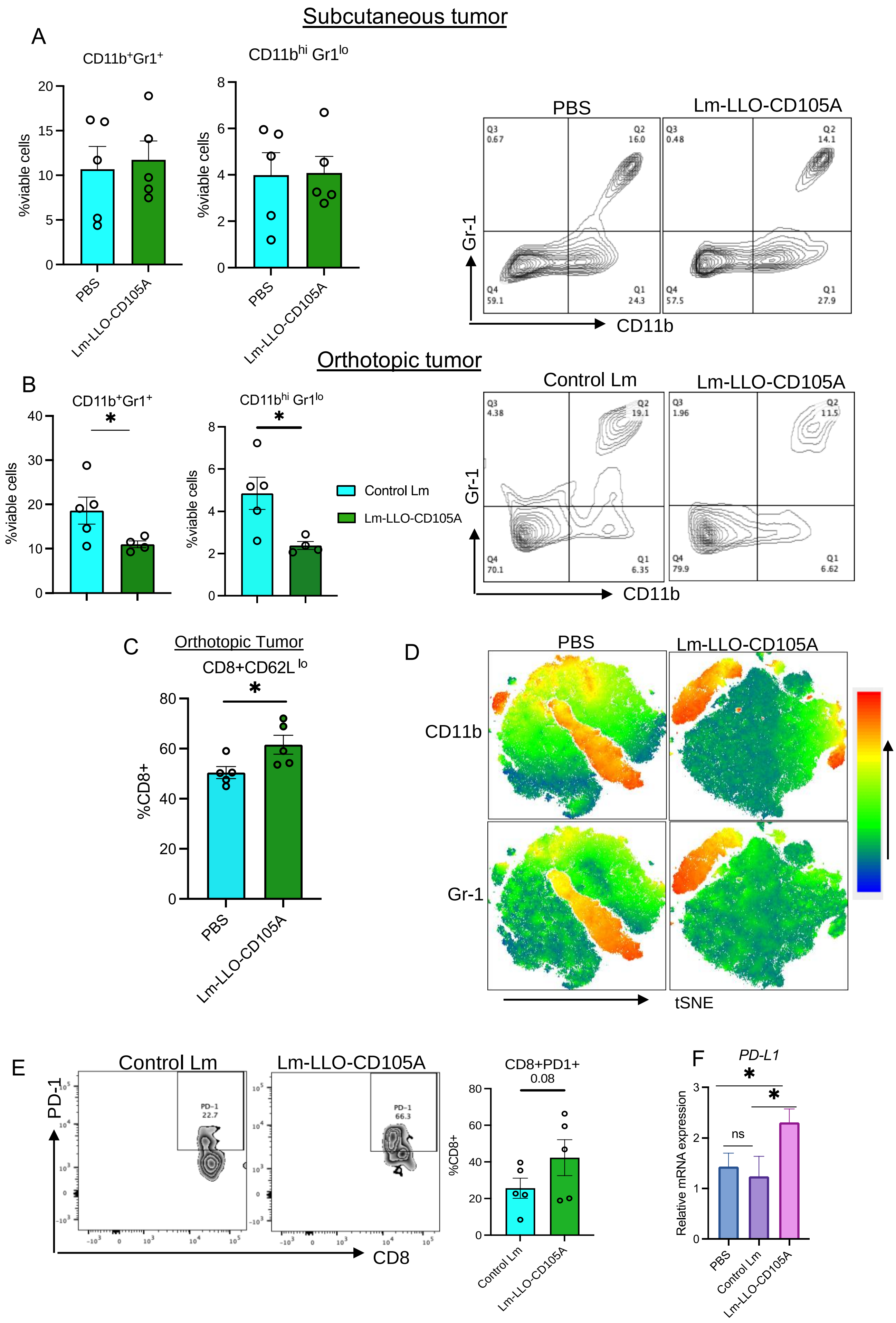

Supplementary Figure S4. Lm-LLO-CD105A reduces MDSCs and moderately increases PD-1/PD-L1 expression (A) MDSC population in the subcutaneous tumor between treatment groups. **(B)** MDSC population in the orthotopic tumor between treatment groups. **(C)** $CD8^{+}CD62L^{lo}$ effector $CD8^{+}$T cell population between placebo and Lm-LLO-CD105A treatment groups in the orthotopic model **(D)** tSNE plot of $CD11b^{+}$ and $Gr\text{-}1^{+}$ cells (**E**) PD-1 expression by flow cytometry and **(F)** *Pd-l1* mRNA expression by tumors from Control Lm and Lm-LLO-CD105A treatment groups (n=5/group). Data were analyzed using unpaired t-test. *$P < 0.05$. All error bars are shown as mean $\pm$ SEM.

Supplementary Figure S5

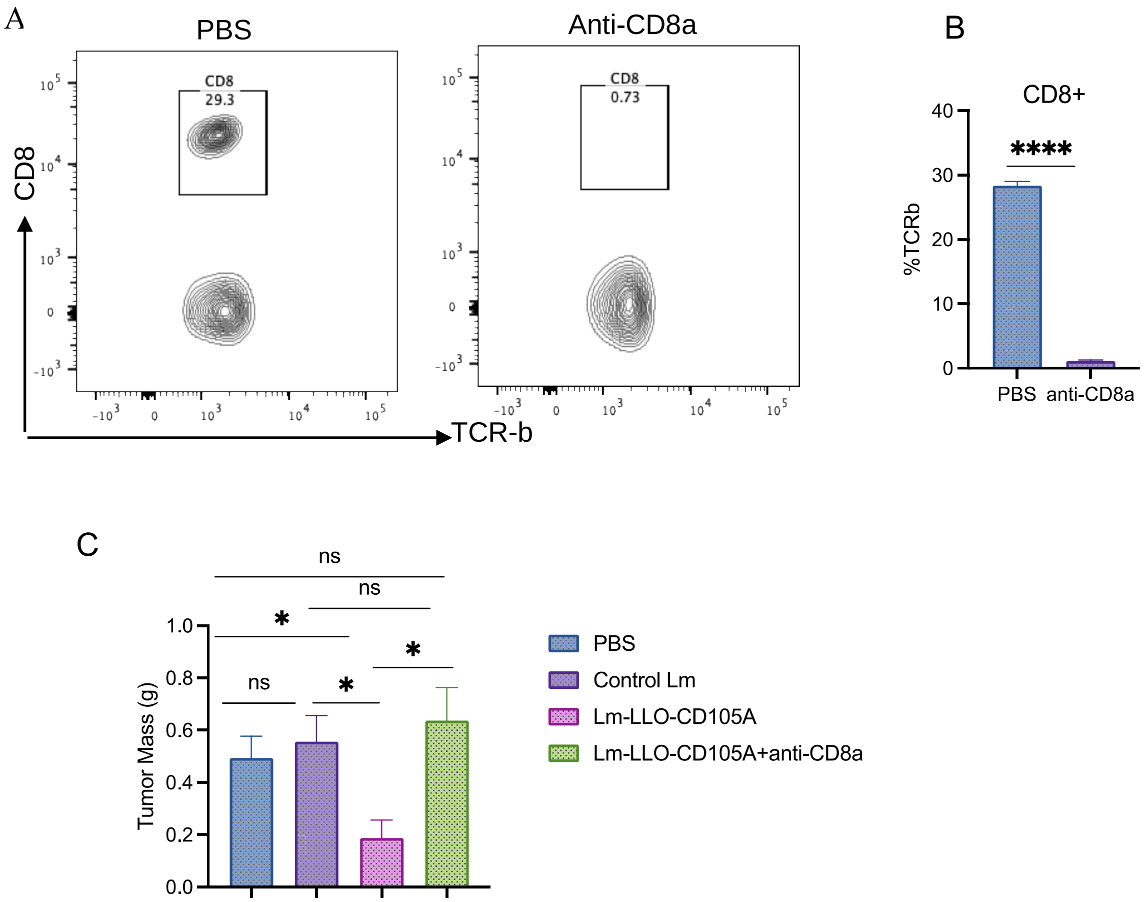

Supplementary Figure S5. Confirmation of $CD8^+$ T cell depletion. (A) Representative plots showing confirmation of CD8 T cell depletion. **(B)** Statistical representation of $CD8^+$ population in the spleen (n=3/group). **(C)** Final tumor mass after CD8 depletion experiments. (n=4-5/group) Data were analyzed using unpaired t-test. *P < 0.05, ****P< 0.0001 ns, not significant. All error bars are shown as mean ± SEM.

Supplementary Figure S6

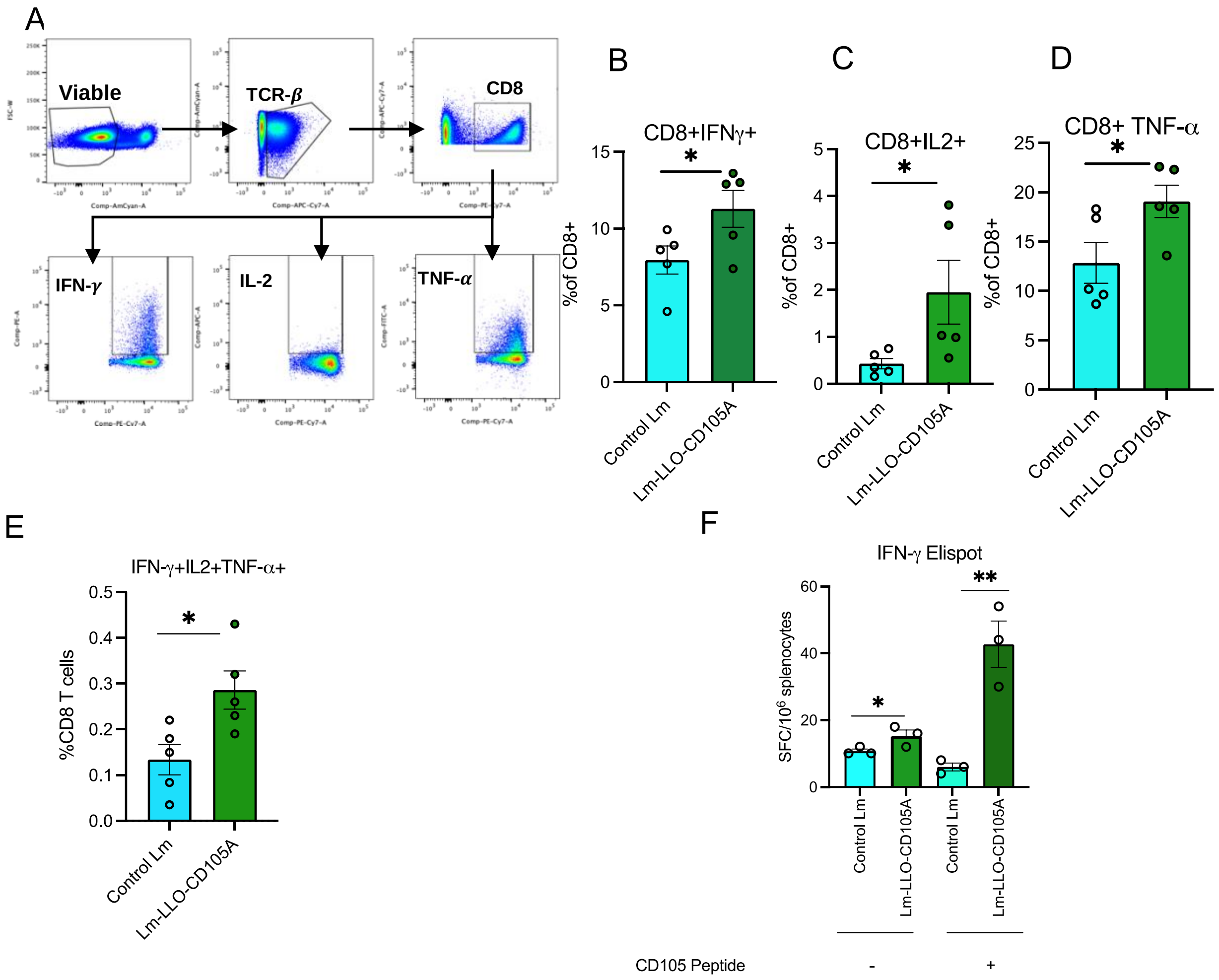

Supplementary Figure S6. Lm-LLO-CD105A increased the polyfunctionality of antigen-specific CD8^{+} T cells in the spleens. (A) gating strategy for CD8^{+} T cells, and cytokine production. Production of **(B)** IFNg **(C)** IL-2 and **(D)** TNF-a by the splenic CD8^{+} T cells after treatment with Lm-LLO-CD105A or Control Lm. **(E)** IFNg^{+}IL2^{+}TNFa^{+} CD8^{+} T cell population in the Lm-LLO-CD105A and Control Lm treatment groups (n=5/group). **(F)** IFN-γ Spot forming cells between Control Lm and Lm-LLO-CD105 . *P < 0.05 **P< 0.01 All error bars are shown as mean ± SEM.

Supplementary Figure S7

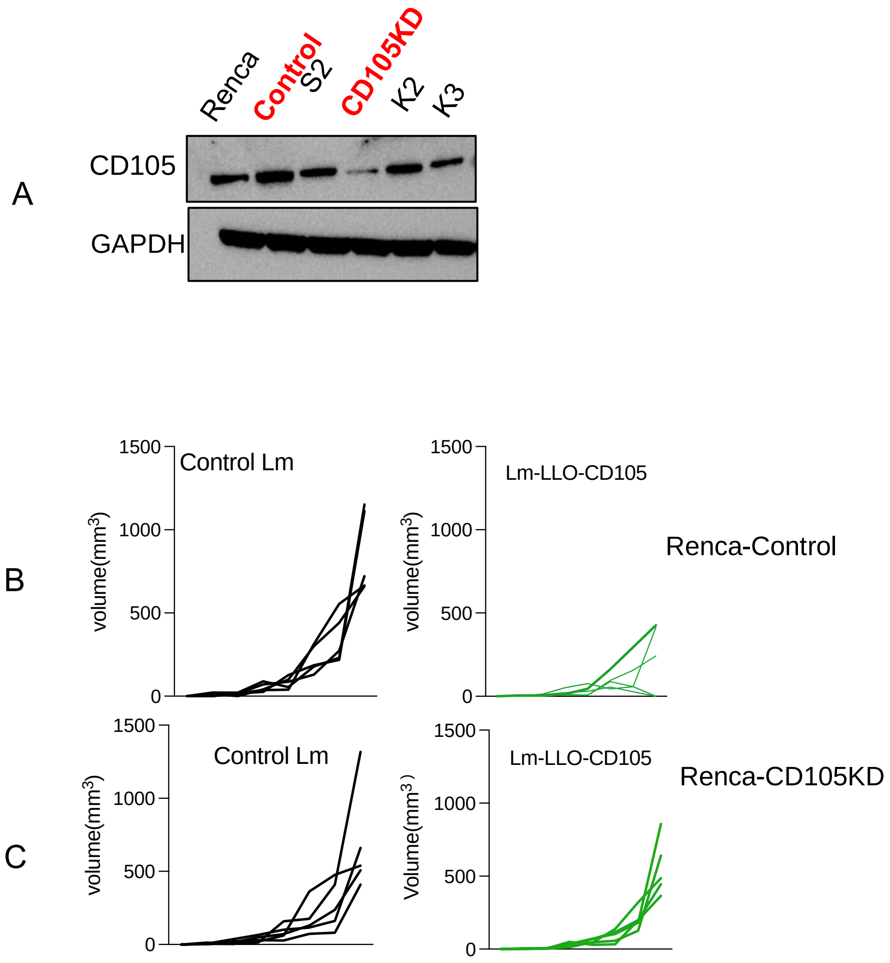

Supplementary Figure S7. Ablation of CD105 abrogates Lm-LLO-CD105A's therapeutic efficacy (A) Western blot confirmation of CD105 depletion in Renca cells. **(B-C)** Individual tumor growth curve of **(B)** Renca-Control and **(C)** Renca-CD105KD after vaccination with Lm-LLO-CD105A or Control Lm (n=5/group).
